# Investigating mechanical deformation’s role in cochlear implant durability

**DOI:** 10.1371/journal.pone.0306613

**Published:** 2024-07-09

**Authors:** Tatiana Blank, André Marcel Ahrens, Christian Klose, Demircan Canadinç, Thomas Lenarz, Hans Jürgen Maier

**Affiliations:** 1 Institut für Werkstoffkunde (Materials Science), Leibniz Universität Hannover, Garbsen, Germany; 2 Department of Mechanical Engineering, Koç University, Sarıyer/İstanbul, Turkey; 3 Department of Otorhinolaryngology, Hannover Medical School, Hannover, Germany; University of Sharjah, UNITED ARAB EMIRATES

## Abstract

Platinum and platinum-based alloys are used as the electrode material in cochlear implants because of the biocompatibility and the favorable electrochemical properties. Still, these implants can fail over time. The present study was conducted to shed light on the effects of microstructure on the electrochemical degradation of platinum. After three days of stimulation with a square wave signal, corrosive attack appeared on the platinum surface. The influence of mechanical deformation, in particular rolling, on the corrosion resistance of platinum was also prominent. The cyclic voltammetry showed a clear dependence on the electrolyte used, which was interpreted as an influence of the buffer in the artificial perilymph used. In addition, the polarization curves showed a shift with grain size that was not expected. This could be attributed to the defects present on the surface. These findings are crucial for the manufacture of cochlear implants to ensure their long-term functionality.

## Introduction

Cochlear implants allow people with severe hearing loss to relearn how to hear, thus giving them a better quality of life. This is achieved by stimulating the auditory nerve through an external speech processor that converts sounds into electrical signals [1]. The first implantable device was developed by William House and Jack Urban in the 1960s, but only had a single electrode. Much has been developed and improved in recent years. Nowadays, a multichannel electrode array is used to better cover the different frequencies [2].

However, some of these implants still fail after long-term service (≈ 20 years) and have to be explanted [3–5]. The inner ear, despite its status as an immune-privileged organ, has frequently exhibited local and systemic inflammatory responses. These inflammatory processes may be involved in compromising the performance of the implant [6]. In addition, fibrous tissue around the electrodes can worsen the impedance and electrical hearing perception of cochlear implants [7]. A key problem with these foreign body reactions is that they often cannot be cured with antibiotics, so that a revision operation with a new implantation is necessary [8].

Depending on the manufacturer, the electrodes are made of platinum or platinum-iridium alloys. Platinum is known for its charge injection capability and stability, making it ideal for long-term applications [9]. In general, platinum has long been popular in biomedical applications, such as treating cancer or inhibiting bacterial proliferation [10]. The widespread use of platinum is due to the metal’s biocompatibility, inertness, high electrical conductivity, and radiopacity [11]. The platinum-iridium alloys feature similar properties but are known for their increased hardness [12]. Although these materials are considered to be inert and corrosion resistant, severe corrosive attack can often be noted on explanted electrodes. These implants were found to be particularly prone to pitting corrosion and surface degradation [13, 14]. The work of Shepherd et al. found that stimulation with high charge densities caused the platinum to corrode [15]. In addition to stimulation, Doering et al. showed that platinum degradation is generally caused by the repeated formation and removal of platinum surface oxide, even within the electrochemical stability window of water. A platinum oxide layer forms on the electrode at potentials above 0.8 V vs. the standard hydrogen electrode (NHE) and decomposes to metallic platinum below 0.8 V vs. NHE. This phenomenon is indicative of surface processes and electrode roughness. Platinum degradation varies with the electrolyte. In phosphate buffered saline (PBS), cycling causes roughening and electrode dissolution, while in H_2_SO_4_, initial roughening and early surface charge loss occurs with electroactive residues. This roughening can lead to electrode dissolution. Lower pH and the widening of the potential window contributed significantly to the degradation [16]. In addition, other studies have found that corroded platinum increases the impedance of the cochlear implant, indicating technical problems or inflammatory reactions. The study by Wissel et al. examined the effect of platinum on the metabolism of mouse fibroblasts and human neuroblastoma cells. It was found that platinum concentrations of 100 mg/ml resulted in a significant loss of metabolic activity and swelling of mitochondria. This is indicative of cytotoxicity. Thus, in addition to the technical problems associated with the corrosion of platinum, it can also lead to cell damage [14].

The stimulation parameters used for a cochlear implant, certainly govern the degradation behavior. However, studies of explants hinted at microstructural effects such as grain size, dislocation density or presence of impurities that could also be key factors in this context. Cochlear implants often feature electrodes made from thin rolled foils with unspecified rolling and heat treatment conditions. The extent to which deformation upon rolling and heat treatment affect platinum is not yet known. The situation is, however, different for other biocompatible materials, where it was shown that use of appropriate rolling parameters and heat treatment can result in grain refinement and affect corrosion resistance. For instance the strength of the magnesium alloys WE43 is reported to increase as the rolling temperature increases, and tailored heat treatments result in improved corrosion resistance [17]. Other materials, such as cold rolled tantalum/tungsten alloys also show significantly less dissolution than the undeformed reference samples [18]. Thus, better understanding the microstructure-process-property relationship could be an avenue to enhance the long-term service behavior of platinum electrodes used in cochlear implants. As a first step in that direction, the present study was designed to shed light on the corrosion behavior of platinum in the rolled as well as in the heat-treated condition.

## Material and methods

### Microstructure

Platinum foils with an initial thickness of 1 mm were used for the rolling. Due to their small dimensions (10 mm x 10 mm x 1 mm), the platinum foils were initially clamped for better handling between two stainless steel (1.4303) sheets. The rolling was then performed in 15 passes with an accumulated total strain of 3.7. Prior to the final rolling pass, the stainless steel sheets were removed.

Samples were analyzed in the (i) cold rolled condition, (ii) the cold rolled and then heat-treated condition (1000°C for 24 hours in an evacuated glass tube) and (iii) the hot rolled condition. These conditions were selected based on the work of Salanov et al., where the platinum was recrystallized between 1000 K and 1400 K. The best results were obtained at the highest temperature [19]. For the hot rolling the platinum foil in between the stainless steel sheets was placed in an oven at 1000°C for 10 minutes and then rolled. Between each rolling pass, the sample was returned to the oven at the same temperature and for the same duration. The parameters used for rolling are summarized in Table 1.

**Table 1:** Parameters used for rolling of the platinum foils.

Rolling condition	Rolling temperature	Heat treatment
Cold rolling	RT	-
Cold rolling + heat treatment	RT	1000 °C, 24 h
Hot rolling	1000°C (start of rolling)	1000 °C, 10 min.

RT: room temperature

To determine the grain size and to remove any residual impurities that may have been caused by the process, the rolled samples were polished down to a 1 μm finish with oxide polishing suspension (OP-S). All samples were then cleaned in an ultrasonic bath with acetone and finally rinsed with distilled water.

For the analysis of the microstructure a ZEISS Supra 55VP secondary electron microscope (SEM) was used. The surface morphology was studied employing secondary and backscatter electron detectors. The foils were also analyzed by energy dispersive X-ray analysis (EDX) to probe for impurities present in the as-received condition or caused by the rolling processes. Finally, Electron Backscatter Diffraction (EBSD) was used to determine texture and grain size.

### Electrochemical measurements

The electrochemical studies were performed in a three-electrode configuration at 37°C. As reference electrode a saturated Ag/AgCl-electrode was used. The counter electrode was made of platinum and the rolled platinum served as the working electrode.

The potentiostat used for the investigations was from GAMRY Instruments (type: Reference 600). The artificial perilymph was prepared according to the recipe proposed by Prasad et al. [20]. The buffer HEPES (4-(2-hydroxyethyl)-1-piperazineethanesulfonic acid) was used in the perilymph. The setup used was aerated to ensure a constant oxygen concentration in the electrolyte. All experiments were performed in a Faraday cage to minimize environmental perturbations.

Prior to the measurements, the surfaces were cleaned at room temperature in 0.1 M H_2_SO_4_ with constant stirring according to Hibbert et al. [21]. A voltage of 2.76 V was first applied. This was repeated three times with a rest period of ten seconds in between. This procedure was then repeated again at -3.24 V. This was followed by a constant increase in voltage in steps of 0.10 V to 0.76 V. After that, five cyclic voltammograms were recorded until they were reproducible. These started at a potential of 0 V with an upper potential limit of 0.96 V, and a lower potential limit of -0.84 V. The scan rate was 0.5 V/s. After the cyclic voltammograms, the cleaning was completed. The entire cleaning regime was repeated ten times. The cleaning regime is summarized in Table 2.

**Table 2:** Parameters for the cleaning regime.

	*E*_lower,_ V	*E*_upper,_ V	Scan rate, V/s
1. Step (3 times, rest period 10 s)	2.76	-	-
2. Step (0.1 V steps)	-3.24	0.76	-
3. Step CV (5 times)	-0.84	0.96	0.5

### Polarization measurements

Polarization measurements were conducted to determine the corrosion rate and the data was analyzed using Tafel plots. For these measurements, the potential was varied between -0.1 V and 0.4 V at a scan rate of 1 mV/s. The measurement points were recorded every 0.5 s and care was taken to ensure that the results stabilized before a measurement was completed.

### Cyclic voltammetry

For the cyclic voltammetry measurements, both the artificial perilymph and an 0.1 M H_2_SO_4_ were used. The latter was employed as a reference medium to probe the sensitivity of the system to a drastic change in the environmental conditions. In case of the 0.1 M H_2_SO_4_, a lower potential limit of -0.3 V and an upper potential limit of 1.3 V were selected with a scan rate of 0.5 V/s. The same scan rate was used for the measurements in artificial perilymph. However, the lower potential limit was set as -1.1 V and the upper potential limit was 1.4 V.

Due to the standard potential of platinum (+1.2 V), a voltage value of at least 1.2 V in the positive direction had to be selected. A potential window of -1.0 V to +1.2 V against Ag/AgCl was then used for the start. This range covers the stability of platinum in water and avoids significant oxygen evolution at the positive end and hydrogen evolution at the negative end. The final potential values were then gradually approached. The parameters used for cyclic voltammetry and polarization measurement are shown in Table 3.

**Table 3:** Parameters for the electrochemical measurements.

Procedure	*E*_lower,_ V	*E*_upper,_ V	Scan rate, V/s
CV (0.1 M H_2_SO_4_)	-0.3	1.3	0.5
CV (perilymph)	-1.1	1.4	0.5
Polarization measurement	-0.1	0.4	0.001

### Stimulation of the foils

**Fig 1:**
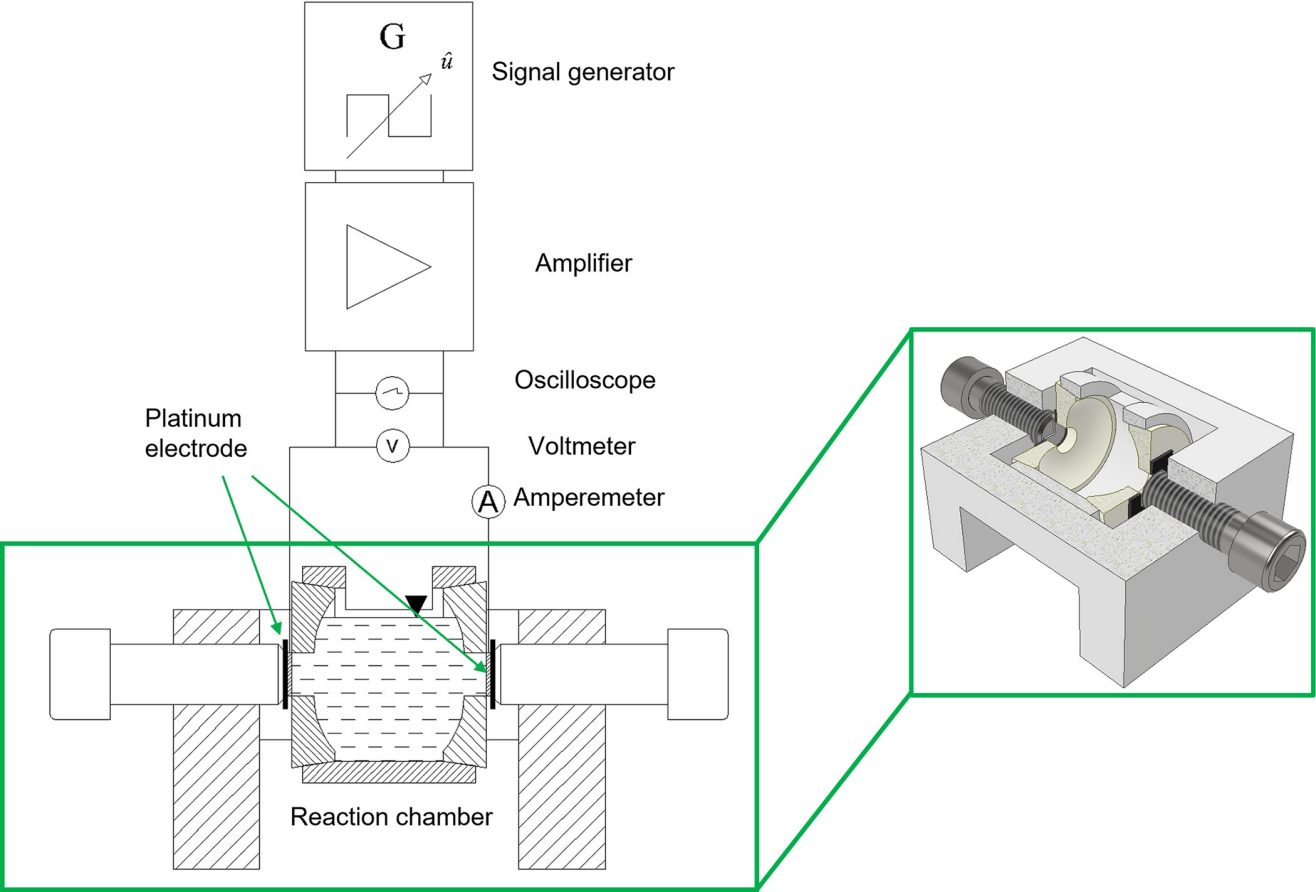
Experimental setup used for electrical stimulation of the platinum foils.

For the electrical stimulation of the platinum foils, an alternating symmetric square wave signal was applied with a signal generator and amplifier at a frequency of 3 kHz. The electrolyte used was a 30 ml artificial perilymph solution, identical to the one used in the electrochemical measurements. The stimulation duration of the experiment was three days. The experimental setup used for electrical stimulation of the platinum foils is shown in Fig 1.

The setup used to stimulate the platinum foils consisted of a reaction chamber and two polytetrafluoroethylene (PTFE) reaction chamber lids, cf. Fig 1. The conical lids were clamped against the reaction chamber using two M14 cylinder head screws in the test rack. The platinum electrodes, which were separated from the screws by insulators, were located between the screws and the reaction chamber lids. This experimental setup allowed the chamber to be completely sealed and filled with electrolyte to mimic the in-ear situation.

## Results

### Microstructure characterization

**Fig 2:**
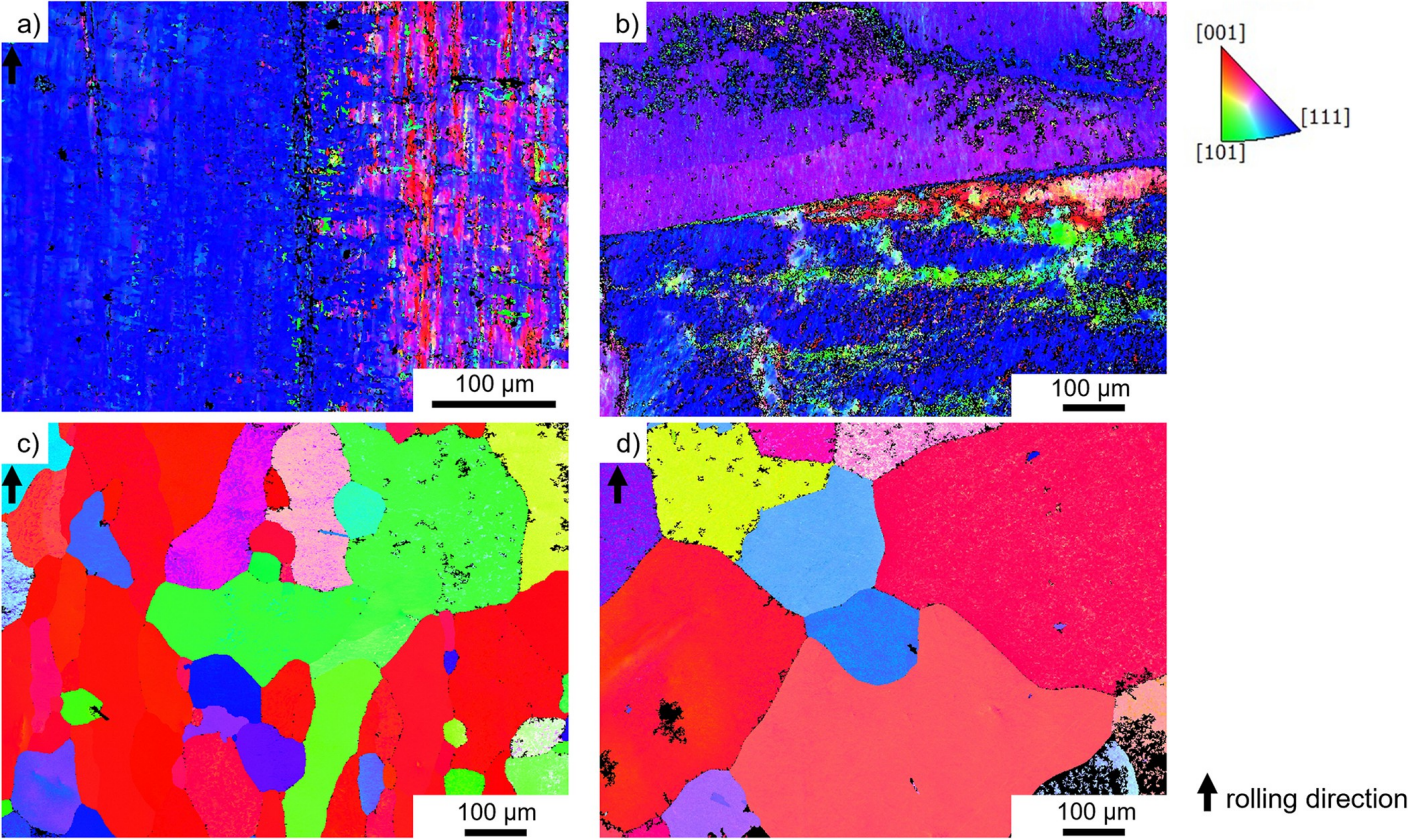
Grain orientation (IPF-X maps). Grain orientation from a) cold-rolled, b) as-received, c) cold rolled and heat-treated, d) hot rolled condition as determined by EBSD; The arrow marking the rolling direction is only valid for a), c) and d), whereas the color-coding provided is valid for all the EBSD maps.

The EBSD images provided in Fig 2 show the grain orientations of the samples after the different rolling tests. The manufacturer did not supply any information about the deformation and heat treatment parameters for the as-received sample. When comparing Fig 2A and 2B it is obvious that both samples feature a pronounced texture in the [111] direction, as indicated by the blue color coding. The image quality maps (not shown) indicated that a high dislocation density is present in both cases. Thus, an accurate determination of grain size was not possible for both the cold rolled and the as-received samples. Moreover, subgrain formation is visible in both Fig 2A and 2B. Thus, the cold rolled state appears to be very similar to the as-received material in terms of microstructure.

After heat treatment, a near-equiaxed microstructure can be seen with a grain size of 140 μm and hardly any texture. Hot rolling brought about a slight additional change microstructure in the form of a slightly increased grain size of 170 μm. Subgrains are no longer present on the mesoscale in both cases and the dislocation density appeared to be low as expected after the high temperature treatments.

**Fig 3:**
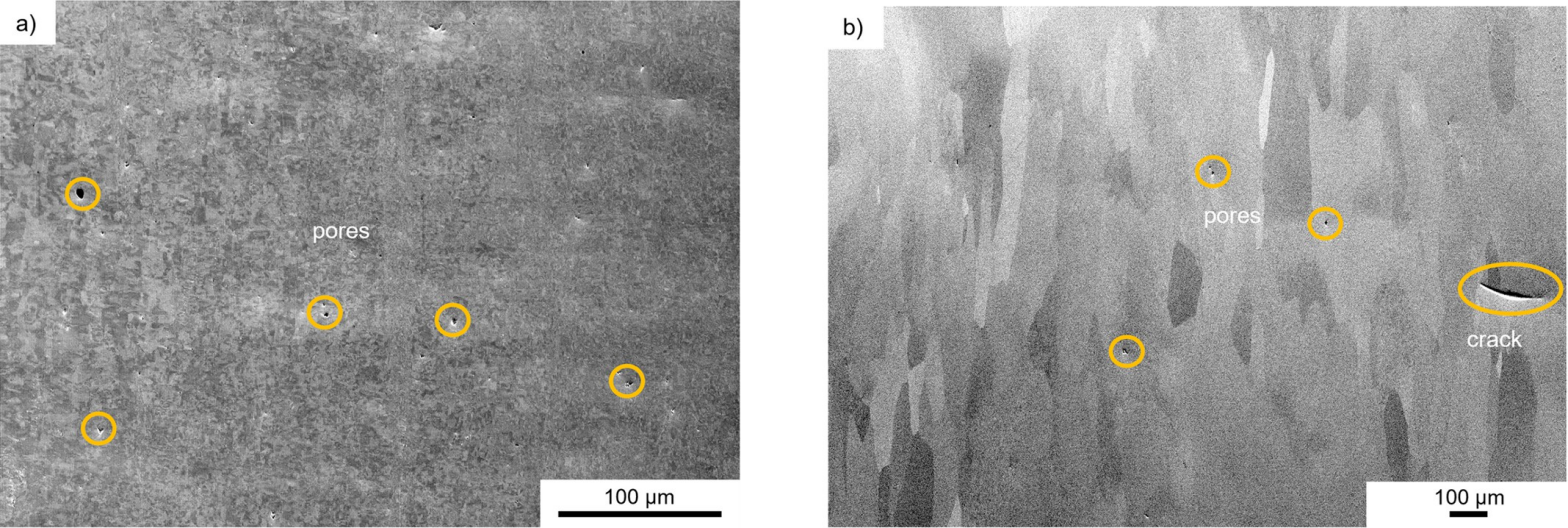
Surface of the rolling samples. SEM images from a) cold rolled and b) cold rolled and heat-treated platinum foil.

When the surfaces were examined with an SEM prior to any of the electrochemical tests, some defects were already noticeable. After cold rolling, defects in the form of pores can be seen on the surface, which are still present after heat treatment, cf. Fig 3. The pores had a diameter of approximately 25 μm. A crack with a length of 250 μm can also be seen on the surface of the heat-treated sample. Similar defects along with scratches that were attributed to handling were also observed on the as-received material.

### Stimulation

**Fig 4:**
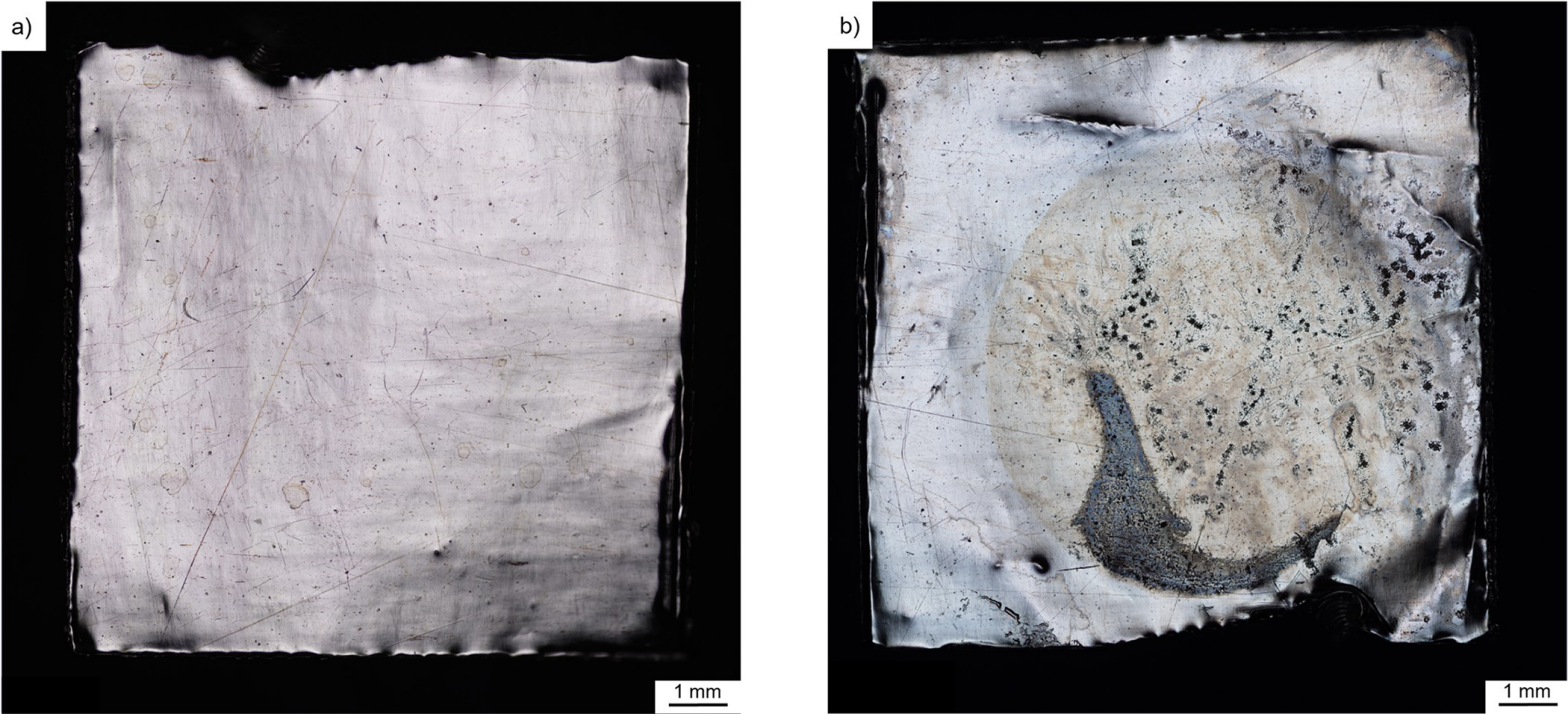
Effects of stimulation on the surface. Platinum foils as-received a) before and b) after three days of stimulation in artificial perilymph.

**Fig 5:**
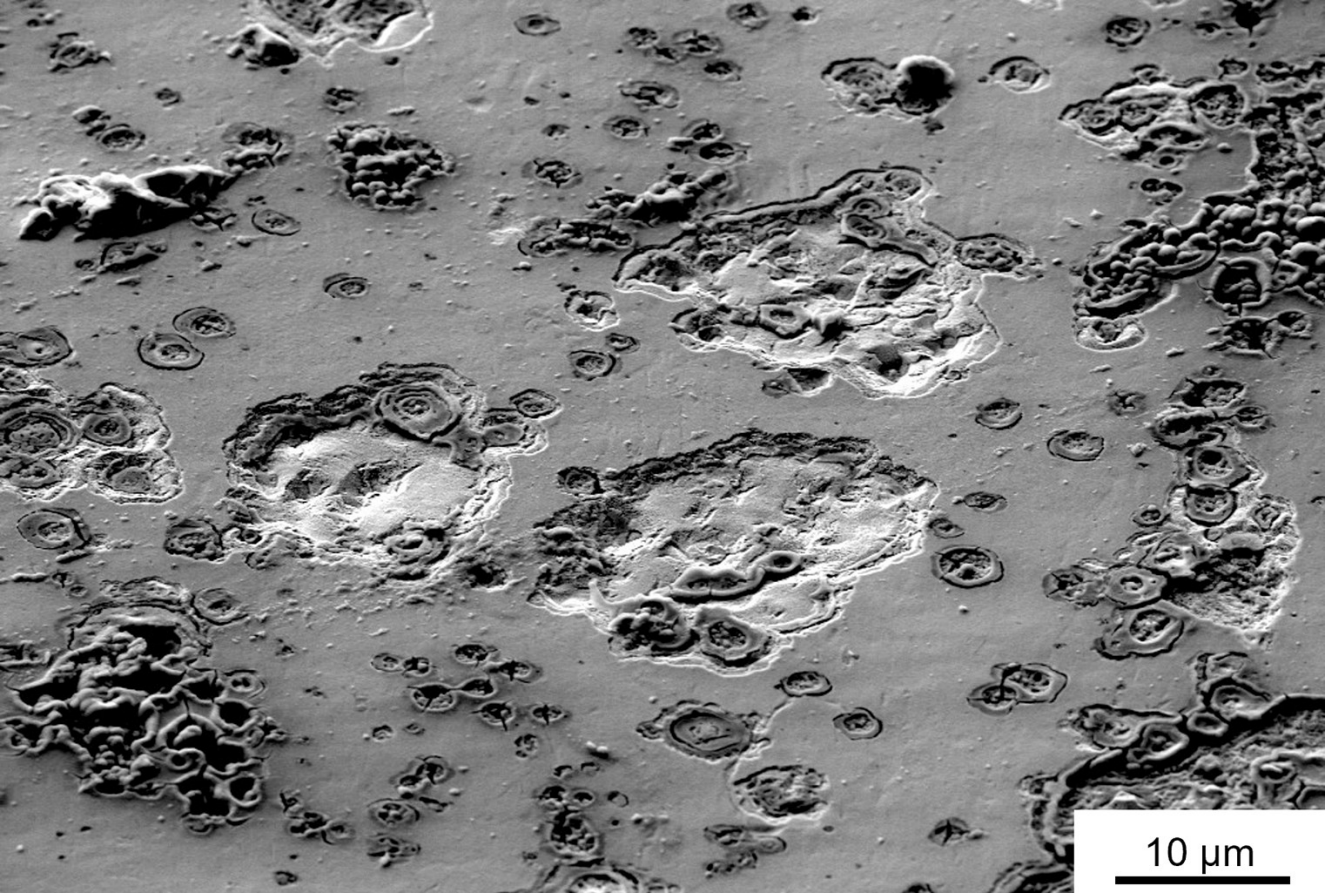
Signs of corrosion on the surface. SEM image after three days stimulation of the platinum foil (as-received) in artificial perilymph.

The stimulation of the as-received platinum foil showed a clear change in the surface, which was already visible with a light microscope at low magnification, cf. Fig 4. Prior to stimulation, the surface looked mostly smooth except for a few small cracks and pores. After stimulation, the pores increased in size and large troughs formed. There is also a slight discoloration of the surface visible in Fig 4B. This sample was then examined by SEM (Fig 5), where the corrosion attack is clearly seen. In addition to deposits on the surface, pitting corrosion can be observed. The troughs formed have a size up to 19 μm. The deposits on the surface were identified as salt compounds, which were part of the artificial perilymph.

The stimulation was conducted over several days to determine if corrosion was detectable. This experiment was performed only on the as-received sample. The comparison of the two samples before and after stimulation (Fig 4) shows that this was the case. Mechanical deformation was assumed to have an additional effect, and this was probed using deformed samples analyzed by electrochemical methods.

### Polarization curves

**Fig 6:**
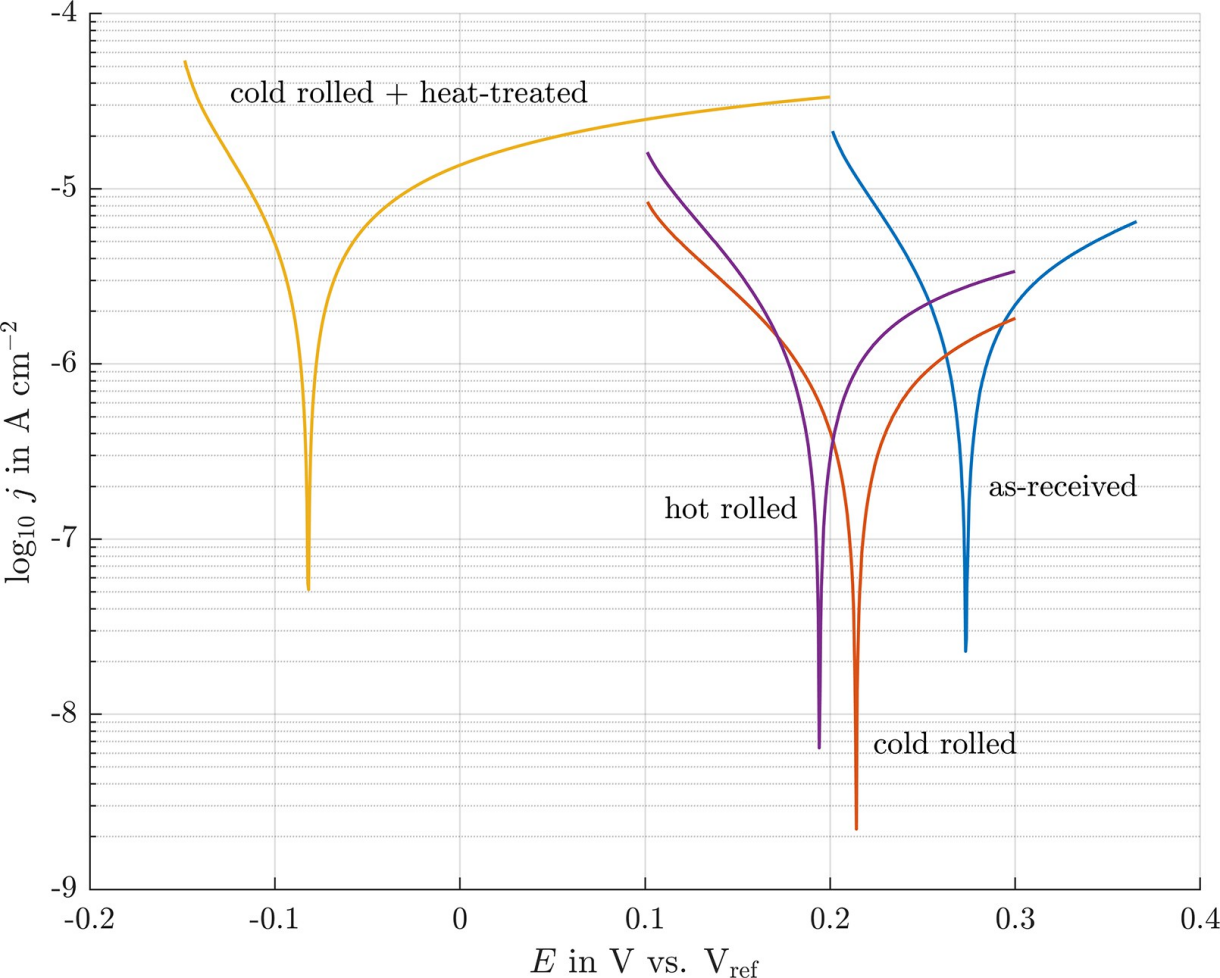
Tafel-Plots of the different platinum foils examined in artificial perilymph.

Clear effects of the material condition are seen in the shift of the corrosion potential and the corrosion current density (Fig 6). The platinum foil in its as-received condition as supplied by the manufacturer had the highest corrosion potential of 274 mV. The corrosion current density around 2 μA/cm^2^ was similar for this and the hot rolled foil. The cold rolled foil showed a slightly lower corrosion current density of around 1 μA/cm^2^. A slight shift towards lower corrosion potentials can be seen. The cold rolled foil shows a slightly higher corrosion potential at 209 mV than the hot rolled sample at 188 mV. The cold rolled foil that was subsequently heat-treated is particularly striking. It shows a strong shift toward a lower corrosion potential of -93 mV and a higher corrosion current density of 8 μA/cm^2^.

### Cyclic voltammetry

**Fig 7:**
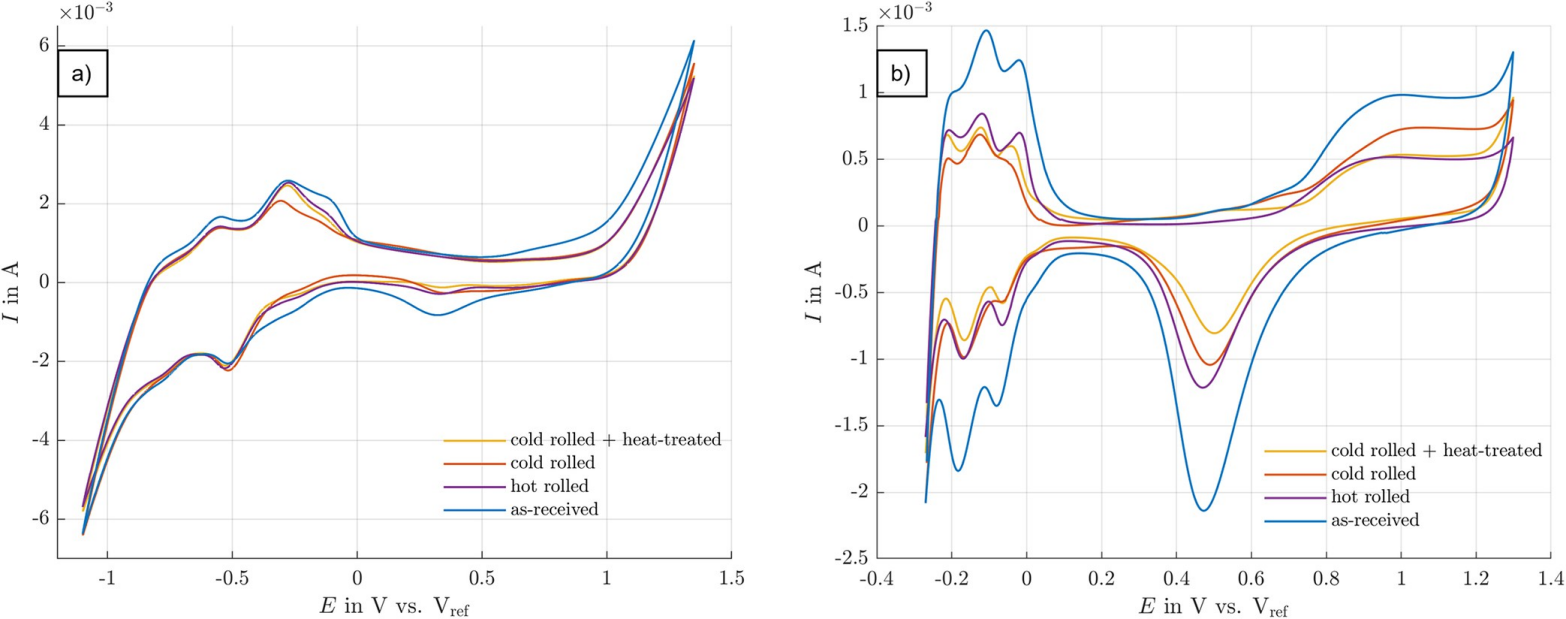
Cyclic voltammograms of the different platinum foils. Measurements were carried out in a) artificial perilymph and b) 0.1 M sulphuric acid.

The measurements in artificial perilymph showed only slight differences between the individual samples. By contrast, the measurements in sulphuric acid demonstrated much clearer differences between the samples with more pronounced peaks in the individual curves (Fig 7).

The most pronounced peaks in sulfuric acid are seen in the as-received platinum foil. The peaks are significantly smaller in the cold rolled and then heat-treated sample. The other two samples show similar characteristics. The cold rolled sample with subsequent heat treatment shows the smallest reduction peak in sulfuric acid and a smaller oxygen oxidation surface. Hydrogen adsorption and desorption are also less pronounced in this sample than in the hot rolled and cold rolled samples without subsequent heat treatment. However, the adsorption and desorption are almost symmetrical in the similar potential range. It is clearly seen that the potential range is different between the two electrolytes. It is wider in the artificial perilymph than in sulfuric acid. The maximum value is about the same, but the lower potential limit is higher in sulfuric acid, i.e. around -0.3 V. A small peak between 0.5 V and 0.3 V can be seen in the cyclic voltammogram from the artificial perilymph, as well as only one clear peak during hydrogen adsorption.

## Discussion

Although no data on the effect of deformation on corrosion behavior is available in the open literature for platinum, this aspect has been widely studied in other materials such as magnesium alloys and part of these findings should be applicable to platinum as well. For instance, in the work of Huang et al. rolling tests were performed on AZ91. It was found that the corrosion resistance is related to the change in grain boundary density after hot rolling. The results showed that the samples without rolling had the best corrosion resistance [22]. One of the reasons for this observation is that the cold-rolled sample has an almost homogeneous microstructure with few grain boundaries. The positive effect of a fine-grained homogeneous microstructure is also apparent in the present study on platinum. When the grains became larger as a result of heat treatment, the higher potential difference led to more pronounced corrosion. Seshweni et al. also found in their study of duplex stainless steel that cold rolling is more suitable than hot rolling for applications where better hardness and corrosion resistance are required [23]. At the same time, the work of Deng et al. and Ma et al. showed the opposite trend. Specifically, Ma et al. investigated the correlation between strength and corrosion resistance in tantalum-tungsten alloys. It was found that grain subdivision was caused by rolling, which resulted in texturization. As a result, electrochemical processes were weakened and corrosion rates were reduced in highly deformed samples [18]. In the work of Deng et al., the WE43 alloy also showed better corrosion resistance in the hot rolled specimens [17].

Based on the EBSD data, the condition of the as-received sample supplied by the manufacturer is similar to that of the cold rolled sample. The similar grain sizes and misorientations indicate that it has also been cold formed, cf. Fig 2A and 2B. The accumulation of dislocations leads to the formation of subgrains, which could be observed in each sample. The thermal post-treatment induced recrystallization, and thus dislocation density is substantially reduced in these samples as well. Thus, one might expect two groups with similar corrosion behavior: (i) the cold rolled and the as-received material (Fig 2A and 2B) and (ii) the two heat-treated variants (Fig 2C and 2D). Contrary to expectations, the current density-potential curves do not reveal the expected trend. All samples showed very similar corrosion current densities and similar corrosion potentials, except for the cold rolled and heat-treated material, cf. Fig 6. Often a positive effect is reported, when smaller grain size leads to a finer distribution of precipitates at grain boundaries, e.g. [17]. Obviously, this is not an issue for the high-purity platinum. Similarly, the pronounced difference in dislocation density is not clearly reflected in the polarization curves as the cold rolled and the hot rolled materials show similar response, cf. Fig 6. Thus, it appears that the number and distribution of surface defects such as pores, cracks or even handling-induced artefacts govern the corrosion behavior. This would explain the approx. five-fold higher corrosion density of 6 μA/cm^2^ in the cold rolled and heat-treated sample, which featured both pores and crack, cf. Fig 3B. This is also in line with the observation that corrosion is a localized phenomenon resulting in the formation of pits (Fig 5). In this context, it is important to note that polarization curves obtained in short-term tests might not reveal the presence of a few local surface defects. In contrast, the much more severe and extended stimulation experiments favor severe localized corrosion attack (Figs 4 and 5). It is speculated that such localized phenomena might explain premature failure of actual cochlear implants. The durability of the heat-treated sample over several days of stimulation was not studied, only the electrochemical response. However, these data showed a clear shift (Fig 6), indicating that the corrosion resistance is significantly worse due to the surface defects that occur (Fig 3B). Consequently, the defects observed in the heat-treated specimen can be expected to cause faster corrosion, if the foils were stimulated for several days.

**Fig 8:**
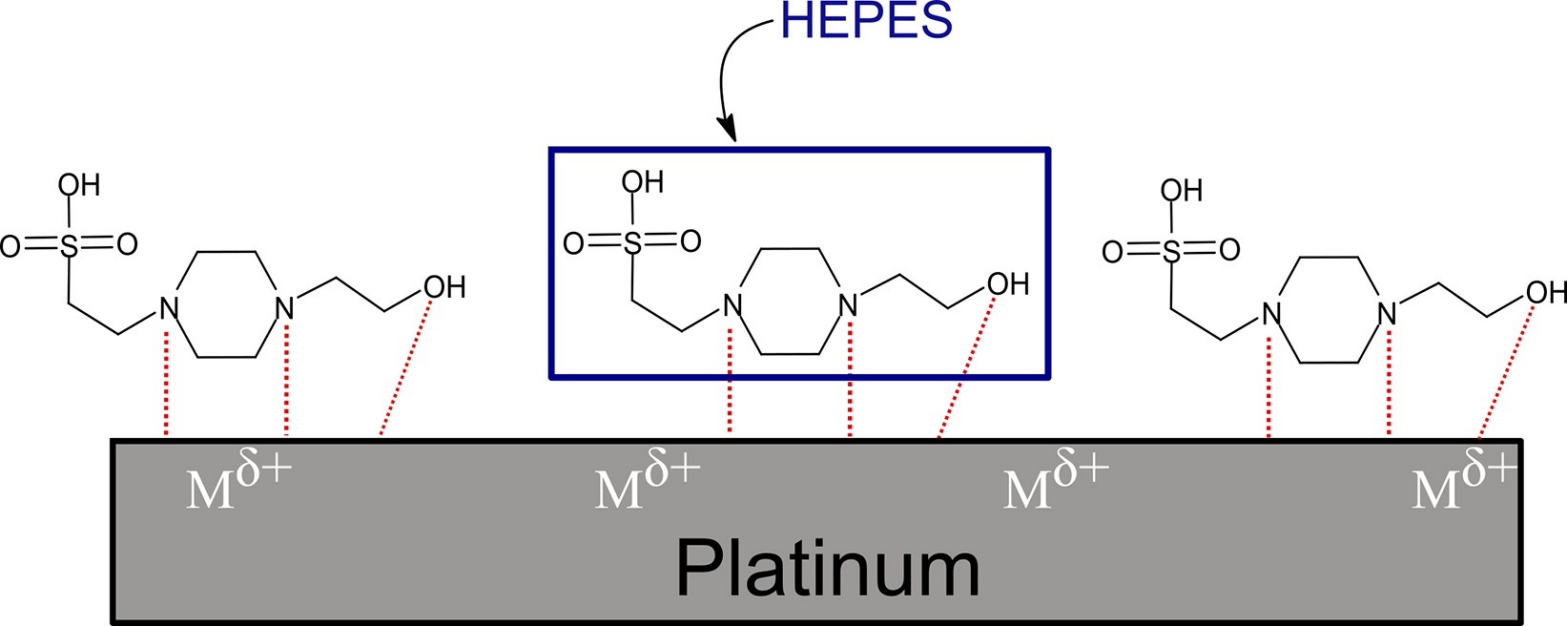
Platinum with HEPES on the surface (according to So et al. [24]).

Another issue that has to be taken into account is the choice of the electrolyte used in the experiments. One often tries to mimic the composition of the perilymph, which is a viable approach for the stimulation experiments. Yet, it does not reveal the differences in reactivity of the various microstructural states, cf. Fig 7A. Here, the HEPES buffer is added to the artificial perilymph to stabilize the pH over time. According to So et al. [24], this molecule is able to bind to the surface of platinum, and thus block the surface as shown in Fig 8.

The work of Michelhaugh et al. [25] investigated such chemisorption of organic molecules on polycrystalline electrodes. They found that the formed monolayer is essential for metal passivation, and thus increases the resistance to electrochemical oxidation. In addition to several other metals, platinum has also been studied. Organic compounds containing at least one surface-active functional group were selected for the investigations. In particular, the strong adsorption of piperazine on the platinum surface, which is also a component of the structure of the HEPES buffer, was emphasized in the work of Dkhili et al. [26]. Stickney et al. also showed that some nitrogen compounds exhibit strong chemisorption, and thus high corrosion resistance [27]. According to El-Shafei et al., the effectiveness of the inhibition depends, among other things, on the number of adsorption sites in the molecule and the size of the molecule. He observed these effects mainly on different amino acids [28]. The HEPES molecule is large and takes up a lot of space, especially with its cyclic moieties. As a result, the platinum surface is shielded and surface reactions are less likely to occur. Thus, the results in artificial perilymph (Fig 7A) all appear similar and the peak between 0.3 V and 0.5 V in the artificial perilymph can be attributed to the complexation of platinum with the chloride ions present in the solution. In particular, [PtCl_4_]^2-^ and [PtCl_6_]^2-^ may be formed [29].

Comparing Fig 7A and 7B clearly show that the measurements conducted in the sulfuric acid reveal the differences between the various samples much better. According to Daubinger et al., the activity of protons or hydroxide ions influences the electrochemical potential. The lower the pH, the higher the electrochemical potential of the electrolyte solution. Thus, reactive processes also take place at a higher potential, as is the case with sulfuric acid, which has a low pH compared to the artificial perilymph [30]. As the cyclic voltammograms are governed by surface reactions and do less respond to local defects such as pores or crack, the effect of increased dislocation density is more prominent in these curves, cf. Fig 7B.

The present results demonstrate that the effects of microstructure, i.e. dislocation density, grain size and texture affect the material response differently. Changes in dislocation density appear to be better reflected in cyclic voltammograms, provided that suitable electrolytes are used, whereas mesoscale defects are better revealed in polarization curves. However, only the stimulation experiments clearly revealed the pronounced localized corrosion attack. It is speculated that the role of surface defects is also decisive in the nucleation of the corrosion pit formation under actual operating conditions of actual implants, and work on explants is underway to address this issue.

## Conclusion

This study investigated the dependence of corrosion of platinum foils on mechanical deformation by rolling and heat treatment to better understand the degradation of platinum electrodes used in cochlear implants. The main results can be summarized as follows:

1. After only three days of stimulation with an alternating symmetrical square wave signal, localized corrosion in the form of pitting occurred.
2. Pores and cracks were found after cold-rolling and were still present after the heat treatment. These surface defects are considered to be critical for the electrodes long-term performance as they can act as preferred sites for localized corrosion attack.
  In short-term polarization curves the pronounced differences in grain size and dislocation density between the samples were hardly seen, as the platinum used was a noble high purity material. Yet, the effects of mesoscale surface defects were obvious resulting in a shift in corrosion potential and an approx. five-fold increase in corrosion current density.
3. The cyclic voltammograms in artificial perilymph were almost independent of the microstructural condition because the surface of the samples was blocked by the HEPES molecule present, and thus many surface reactions were suppressed. By contrast, the experiments conducted in sulfuric acid clearly revealed the effect of microstructure on surface reactivity.
